# FK506 Treatment Prevents Retinal Nerve Fiber Layer Thinning in Organ-Transplanted Glaucoma Patients: A Retrospective Longitudinal Study

**DOI:** 10.7759/cureus.18192

**Published:** 2021-09-22

**Authors:** Valentina Reffatto, Praveena K Gupta, Tamila Williams, Mary E Schmitz-Brown, Gianmarco Vizzeri

**Affiliations:** 1 Ophthalmology, University of Texas Medical Branch, Galveston, USA

**Keywords:** primary open-angle glaucoma, tacrolimus, fk506, neuroprotection, calcineurin

## Abstract

Purpose

This is a retrospective study of primary open-angle glaucoma patients treated with the immunosuppressor FK506 (tacrolimus) after an organ transplant. We assessed whether FK506 might be a potential neuroprotector adjuvant in glaucoma therapy*.*

Patients and methods

Organ transplant patients treated with FK506 for one or more years between 2006 and 2017 at the University of Texas Medical Branch (UTMB) were enrolled. Those selected were patients older than or equal to 50 years of age and had an ophthalmological eye examination with or without diagnostic tests for primary open-angle glaucoma (POAG). Sixty-one eligible subjects were included in the study and matched with the non-FK506 control group for age, gender, race, and follow-up visits.

Results

A lower incidence of POAG was noted in the FK506-treated patients (15%) when compared to the non-FK506 group (22%), though not significant (p=0.34). Among POAG subjects, the average retinal nerve fiber layer (RNFL) thickness decreased at a rate of 1.4 µm per year (p=0.0001) in the non-FK506 control patients versus 0.4 µm per year (p=0.34) in the FK506 patients. The superior and inferior RNFL quadrants in the control non-FK506 group had a thinning of 2.2 µm and 2.3 µm per year, respectively, (p=0.003 and p=0.0001), while in the FK506 patients, there was no significant loss. In addition, RNFL thinning in nasal and temporal quadrant also showed less reduction in FK506-treated subjects but was not statistically significant (p=0.68 and p=0.93).

Conclusion

FK506 therapy offers a new promising avenue for neuroprotection in POAG patients and needs to be investigated further for use in conjunction with conventional glaucoma treatments.

## Introduction

Glaucoma is considered to be one of the leading neurodegenerative disorders characterized by the loss of retinal nerve fiber layer (RNFL) at the optic nerve head due to the death of retinal ganglion cells (RGC) [1]. Although the mechanism underlying RGC death is still not fully understood, evidence suggest that RGCs die as a result of an apoptotic process with consequent chromatin condensation, DNA fragmentation, oxidative damage, and autophagic degeneration [2]. For instance, in patients affected by primary open-angle glaucoma, the presence of inter-nucleosomal DNA fragmentation in the RGCs was found to be elevated 15.2 times compared to healthy eyes [3]. Observations concluded from experimental rodent models of glaucoma with elevated intraocular pressure (IOP) suggest RGC death is an apoptotic type of cell death [4-6].

RGC apoptosis is hypothesized to be initiated by several underlying mechanisms, such as an imbalance in calcium homeostasis, neurotrophin deprivation, glial activation, excitotoxicity, ischemia, or oxidative stress [5]. Among them, dysregulation of calcium homeostasis has been proved to play a critical role in many neurodegenerative diseases [7,8]. Elevation of intracellular calcium leads to the activation of numerous Ca^2+^-dependent enzymes including calcineurin (CaN), a Ca^2+^-activated phosphatase highly abundant in the retina [9] and in the brain [10]. Active CaN causes dephosphorylation of the pro-apoptotic B-cell lymphoma 2 (Bcl2)-associated agonist of cell death (BAD) family member, which triggers cytochrome C release from the mitochondria to the cytosol, activating the caspase family of proteases thereby inducing apoptosis [11,12]. In neurons, hyper-activation of CaN has been associated with apoptosis, neurodegeneration, and synaptic dysfunction [13].

In glaucoma patients, the only known mechanism leading to RGC apoptosis is elevated IOP, and management of increased IOP is at present the only available therapy for glaucoma. However, many patients experience RGC and vision loss despite effective IOP lowering treatment, including normal-tension glaucoma patients who develop glaucomatous features without eye pressure exceeding the normal range [14]. This enforces the theory that factors other than IOP contribute to the development of glaucoma [15]. According to this observation, in the experimental glaucoma rodent model, CaN cleavage is shown to trigger RGCs apoptosis with or without ocular hypertension [16,17].

As a therapeutic strategy, blocking CaN activation may prevent RGC damage that occurs with or without elevation of IOP. Oral administration of the CaN inhibitor FK506 (Astellas Pharma, 2-5-1, Nihonbashi-Honcho, Chuo-Ku, Tokyo 103-8411, Japan; trade names: Tacrolimus, Prograf, Advagraf, Protopic7) has been proven to increase RGC survival and optic nerve preservation in a glaucoma animal model [16], and also to reduce RGC death in an optic nerve crush model [18,19]. Routinely, FK506 is also implemented as immunosuppressive therapy in organ transplant recipients to prevent and treat allograft rejection. In this study, we investigated the neuroprotective role of FK506 in primary open-angle glaucoma (POAG) patients using the immunosuppressant FK506 after an organ transplant.

## Materials and methods

Study design

The study was conducted in compliance with the University of Texas Medical Branch (UTMB) Galveston Institutional Review Board (IRB #17-0053) and adhered to the tenets of the Declaration of Helsinki. This is a population-based, cross-sectional matched study of patients who were seen at the University of Texas Medical Branch (UTMB) between 2006 and 2017 on Galveston Island located in the South East Texas region. A Structured Query Language (SQL) system was used to filter patient data from the Electronic Health Record (EHR) system (EPIC) based on our study criteria.

A total of 20,320 patient charts were screened to find eligible subjects who qualified for our study. Recruited patients who were 50 years or older had comprehensive eye examinations at the UTMB eye clinic and were on oral FK506 for a minimum of one year prior to evaluation due to a history of organ transplant. We identified 285 subjects who had a record of undergoing FK506 treatment. Among them, we excluded 177 patients who had been treated for less than one year or didn’t have a current FK506 prescription, and 47 patients who took topical or systemic cyclosporine as this drug is in the same family of CaN inhibitors. A total of 61 eligible subjects receiving one or multiple organ transplants and systematically treated with FK506 were included in the study. Among them, 39 received a kidney transplant, 15 had a liver transplant, four received both kidney and pancreas transplants, two had a heart transplant, and one subject received a bone marrow transplant. These 61 subjects were matched with a non-FK 506 control group of subjects (1:2 ratio approximately, 125 subjects) using a propensity score matching for age at first and final visits, gender, race, and follow-up visit durations.

**Figure 1:**
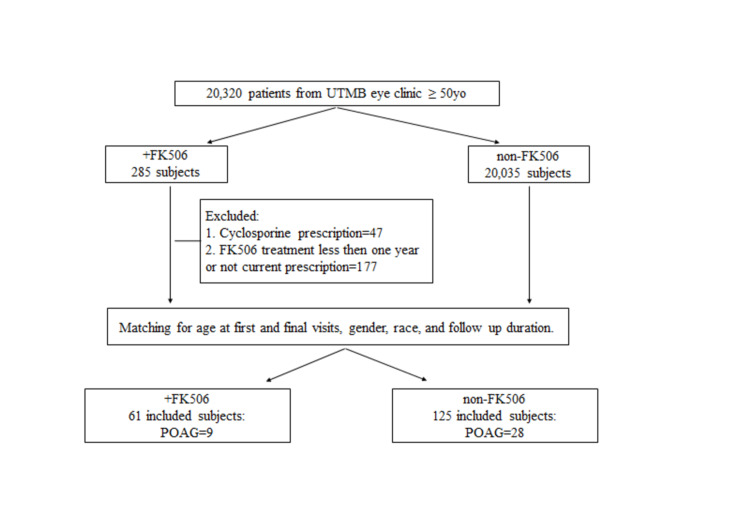
Schematic representation of the study design and patient enrollment UTMB: University of Texas Medical Branch; POAG: primary open-angle glaucoma; FK506: tacrolimus

Ophthalmological reports of the above have identified 37 patients in total who had a diagnosis of primary open-angle glaucoma (POAG) based on the International Classification of Diseases, Ninth Revision (ICD-9) code of 365.11 (Figure 1). POAG involves characteristic optic nerve changes with neuroretinal rim thinning, optic disc cupping, wedge-shaped retinal RNFL loss, and an abnormal visual field (e.g., glaucoma hemifield test outside 99% normal limits and/or corrected pattern standard deviation (CPSD) outside 95% normal limits) in at least one eye. In the case of unilateral glaucoma, the fellow eye was excluded from the study. We collected diagnostic data from all the eligible subjects that included sectoral RNFL thickness extracted from optical coherence tomography (OCT) images, IOP, best-corrected visual acuity, visual field data. All OCT images were obtained using Cirrus HD-OCT 4000 (Carl Zeiss Meditec, Germany) or Spectralis OCT (Spectralis; Heidelberg Engineering, Germany) performed by a UTMB Ophthalmology technician, blind to the study. The cut-off for included tests was set to a signal strength equal to or greater than 7. Visual field tests were conducted using the ZEISS Humphrey Field Analyzer 3 (HFA3, Carl Zeiss Meditec AG), central testing 24-2. We included test with reliability indices, false positive, false negative, and fixation loss errors set at less than 10%.

Statistical methods

A propensity score matching (PSM) was used to select a non FK506 control group. For each of the patients in the FK506 group, two non-FK506 control subjects were matched taking into consideration age at the first and final visits, gender, race, and follow-up duration.

Agreement between the FK506-treated group and the non-FK506 control group was assessed by comparing the demographic variables using the Mann-Whitney test for categorical variables or chi-square test for numerical variables. Incidence of glaucoma with relation to treatment group (non-FK506 control versus FK506-treated group) was modeled by logistic regression. RNFL thickness in POAG patients was reported separately for average and locations: superior (S), inferior (I), temporal (T), and nasal (N). RNFL thinning over time was modeled by mixed linear regression with relation to treatment group (control versus treated), and statistical analyses were performed using R statistical software [20]. In all analyses, alpha was set at 0.05 for a 95% level of confidence.

## Results

Demographic and clinical data

Data retrieved from the medical records of 61 subjects who were taking FK506 for a minimum of one year, and 125 matched non-FK506 control subjects were analyzed as below. The mean age of the subjects at the first visit in both cohorts was 60 years (SD ± 7.5; range, min 46.8-max 84.7). The majority of patients (76%) were male and racially subjects were distributed as Caucasians (44%), African American (24%), Hispanic (23%), Asian (7%), and American Indian (2%).

The mean follow-up time for the study period was 2.5 years (SD ± 1.9; range, 0.0-5.7 years) for the non-FK506 control group and 2.8 years (SD ± 1.8; range, 0.0-5.8 years) for the FK506 group. The average duration of FK506 treatment was 4.8 years (SD ±1.5; range, 1.0-6.7 years), with a dose of 3.8 mg per day (SD ± 1.9mg/day). Data are reported in Table 1.

**Table 1:** Demographics of the study population FK506: tacrolimus ^a^Mann-Whitney test. ^b^Chi-square test.

	Non-FK506 control n=125	FK506 n=61	p-Value
Age at first visit, mean ± standard deviation	60.0 ± 7.6	59.9 ± 7.3	0.37^a^
Age at last visit, mean ± standard deviation	62.6 ± 7.6	61.7 ± 7.1	0.44^a^
Gender			
Male	75%	78%	0.71^b^
Female	25%	22%	0.71^b^
Race (%)			
Black/African American	24%	25%	0.97^b^
Caucasian/White	46%	41%	0.97^b^
Hispanic/Latino	22%	24%	0.97^b^
Asian	6%	8%	0.97^b^
American Indian/Alaskan Native	2%	2%	0.97^b^
Follow-up (years), mean ± standard deviation	2.6 ± 1.9	2.8 ± 1.8	0.39^a^

Primary open-angle glaucoma incidence

POAG incidence was calculated in both the FK506 and in the non-FK506 control cohorts. Analysis using logistic regression showed that the incidence of glaucoma among the cohort treated with FK506 was lower, but not statistically different (p=0.34) when compared to the control. Out of the 125 subjects in the non-FK506 control group, 28 had diagnosis of bilateral POAG, equal to the 22% of the population, but only 15% (n=9) of the subjects treated with FK506 had the diagnosis.

Glaucoma progression analysis

Data for intraocular pressure (IOP), cup-disc ratio (C/D), corrected visual acuity (VA), and visual field index (VFI) was subsequently collected from subjects with glaucomatous features from both the cohorts. Clinical data were extracted from the records on the UTMB EPIC system from a total of 126 visits performed from 2006 to 2013 and were reported separately for the right and left eye. As shown in Table 2, there was no statistical difference between the two groups with respect to the studied parameters. The average IOP range was between 15.64 and 17.85 and the difference between the FK506-treated group and the non-FK506 control group was not significant (oculus dexter {OD}: oculus sinister {OS} p=0.49). C/D ratio mean didn’t statistically differ between the two cohorts (OD p=0.66:OS p=0.11), and its values varied between 0.54 and 0.70. Visual acuity appeared to have no statistical differences when comparing the two groups with an average of 20/30 vision in both eyes for the FK506-treated group, and of 20/30 in the right eye and 20/25 in the left eye for the non-FK506 control group (OD p=0.87:OS p=0.24). VFI ranged between 88.17 and 93.58, and although a relatively large difference was seen, it was not statistically different (OD p=0.34:OS p=0.45).

**Table 2:** Clinical data of glaucomatous subjects IOP: intraocular pressure; C/D: cap disc ratio; SD=standard deviation; OD: oculus dexter; OS: oculus sinister The visual field index was automatically calculated by the ZEISS Humphrey Field Analyzer 3 software. ^a^T-test.

	Non-FK506 eye=56	FK506 eye=18	p-Value^a^
IOP			
OD Mean ± SD	17.11 ± 4.6	15.64 ± 4.4	0.14
OS Mean ± SD	17.85 ± 5.1	17.04 ± 6.5	0.49
C/D ratio			
OD Mean ± SD	0.59 ± 0.2	0.55 ± 0.2	0.66
OS Mean ± SD	0.70 ± 0.2	0.54 ± 0.2	0.11
Visual Acuity			
OD Mean	20/30	20/30	0.87
OS Mean	20/25	20/30	0.24
Visual Field Index %			
OD Mean ± SD	91.17(8.4)	93.58(5.64)	0.34
OS Mean ± SD	88.17(22.86)	90.62(11.33)	0.45

Retinal nerve fiber layer (RNFL) thickness was also extrapolated from the OCT images of the optic nerve as a glaucoma progression marker separately for both average and isolated quadrants. A statistical model of mixed-linear regression, relating RNFL thickness to time, showed a statistically significant trend of reduced rates of RNFL thinning for the FK506 patients compared to the non-treated group.

**Figure 2:**
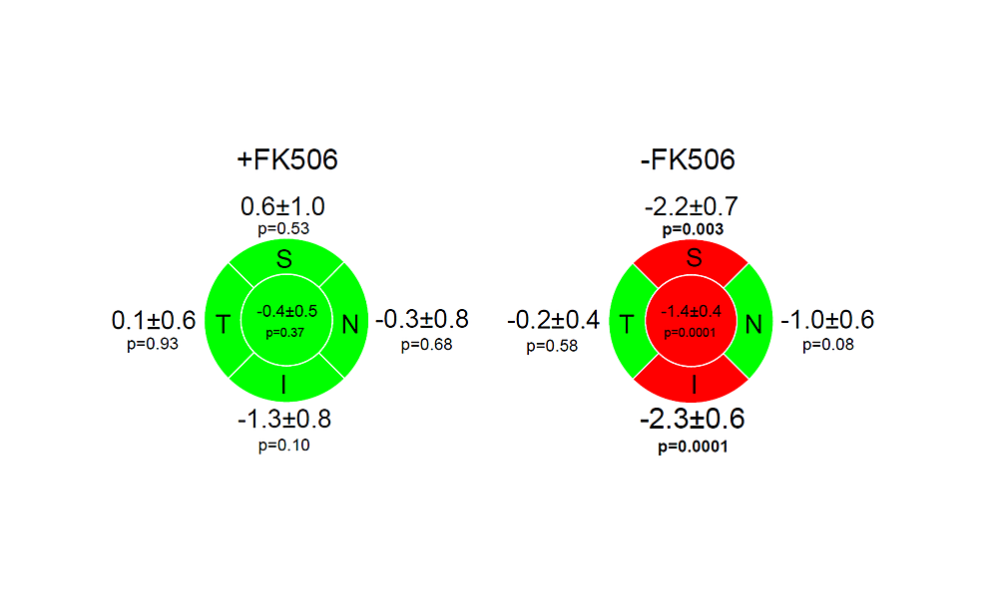
Regression summary for RNFL thickness separated by location S, I, N, T, and average FK506: tacrolimus; RNFL: retinal nerve fiber layer; S: superior; I: inferior; N: nasal; T: temporal The values indicate the change in µm per year ± standard deviation. The green color represents no statistically significant change and the red color represents statistically significant thinning. The p-value cut-off for significance was ≤ 0.05.

**Figure 3:**
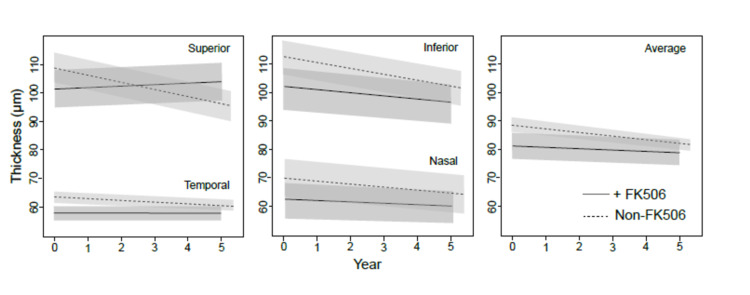
RNFL thickness plotted against time in glaucoma patients treated with FK506 (continuous line) versus non-FK506 (discontinuous line) over five years FK506: tacrolimus; RNFL: retinal nerve fiber layer RNFL thickness is shown separately for all four quadrants and when averaged.

As summarized in Figure 2 and Figure 3, the rate of RNFL thinning in the superior quadrant in the non-FK506 group showed a significant thinning of 2.2 per year (p=0.003), while the FK506 group showed no significant evidence of thinning (p=0.53). Similarly, at the inferior quadrant, the rate of RNFL thinning for the non-FK506 group was 2.3 per year (p=0.0001), while the treated group stayed stable. RNFL thinning in nasal and temporal quadrants showed a tendency to be ameliorated in patients receiving FK506, although it was not statistically significant. Overall, the average RNFL thinning was statistically higher in the non-FK506 group with a rate of 1.4 µm per year (p=0.0001) compared to the FK506 group which showed a thinning rate of 0.4 µm per year (p=0.34).

## Discussion

Glaucoma is a form of neurodegenerative disease and follows cell death pathways similar to Alzheimer's (AD) and dementia. Several studies have elucidated common molecular mechanisms underlying the pathogenesis of these conditions. CaN activation with consequent BAD dephosphorylation and Cytochrome C release are implicated in initiating neuronal cell death in several neurodegenerative diseases including AD [21], Parkinson’s disease (PD) [22], and Huntington’s disease (HD) [23]. FK506 is a CaN inhibitor with neuroprotective properties with promising results in memory and learning improvement in the AD mouse model [24], and with protective effects on experimental spinal cord injury [25], PD [26], and HD [27].

In this retrospective observational study, we evaluated the effect of the CaN inhibitor FK506, on the incidence and progression of primary open-angle glaucoma. We compared subjects treated with FK506 with a non-FK506 control group and followed them between two and five years. To avoid potential bias the two cohorts were matched for age at the first and final visit, year of first and final visit, gender, and race. IOP also did not differ between the FK506 group and the non-FK506 control, as all POAG subjects in both groups were treated with IOP lowering drops. The incidence of POAG was 7% lower in subjects treated with FK506, even though it was not significant from those of the non-FK506 control group. In the current study, we only included patients that had salient glaucomatous damage and excluded suspects who could potentially convert to glaucoma in near future. Therefore, a future study on the use of FK506 in arresting glaucoma progression in patients that are glaucoma suspects is a plausible hypothesis.

Measurements of RNFL thickness as a marker of POAG progression showed a significant decrease in RNFL loss over time among patients receiving FK506 versus the non-treated group. A marked decrease in the RNFL thinning was noted in the inferior and superior quadrant in the group of patients receiving FK506. This finding is particularly exciting as it is well documented that glaucomatous optic nerve damage follows a pattern where inferior and superior quadrants undergo the greatest amount of neuro-retinal rim thinning. In a normal eye, the neuro-retinal rim is thickest inferiorly, followed by the superior and nasal rim, and thinnest temporally. This pattern is known as the ISNT rule (i.e., inferior, superior, nasal, temporal). However, in glaucomatous patients, we generally observe vertical thinning with atrophy along the inferior and superior rim, with a deviation from the ISNT rule. Overall, combined, the average RNFL also has a lower thinning rate in the FK506-treated group. Whether this neuroprotective effect can stop the progression of glaucoma and prevent visual field loss is perhaps an over prediction of the observation from this study given the small sample size and the short duration of treatment.

To our knowledge, only one large population-based study has looked at the effect of FK506 on the incidence of AD or dementia in solid organ transplant patients. This study revealed a significant reduction of diseases prevalence in the FK506-treated patients [28]. The role of FK506 in AD patients brings new frontiers for glaucoma study, not only because of the high rate of comorbidity reported between AD and glaucoma but also because of the similarity in the structure, function, and pathogenesis of the two diseases [29,30].

The authors are aware of the limitations of this small-scale pilot observational study. We relied on information taken from medical records, which sometimes may not be complete with respect to our interests. Although RNFL thickness seen on OCT indicated the tendency of thinning, especially in the control group, RNFL thinning does not necessarily indicate the presence of a clinical disease unless conjugated with other parameters like visual field (VF) or cupping. For instance, RNFL thinning has been reported in both glaucoma and non-glaucomatous optic neuropathies and central nervous system diseases such as ischemic optic neuropathy, optic neuritis, traumatic optic neuropathy, and degenerative diseases. The present study also does not include RNFL measurement prior to the FK506 treatment start, and further studies are needed to compare the RNFL rate of thinning with and without treatment.

## Conclusions

In conclusion, our findings show for the first time in humans that FK506 may have a protective effect in reducing RNFL thinning in POAG patients. A major drawback of FK506 is that it is a potent immunosuppressive drug and may cause side effects. We were able to disregard the expected side effects because of the comorbid conditions of glaucoma and organ transplant in the subjects receiving FK506. Glaucoma is a challenging condition especially due to the lack of effective systemic treatment to prevent retinal ganglion cells degeneration. Advances in the development of neuroprotective therapy will open new opportunity in the treatment of not only glaucoma patients but also many similar neurodegenerative age-related conditions. Further studies may be pursued to titrate a clinically acceptable dose of FK506 that can provide a neuroprotective arm in the therapy of glaucoma.
